# Supplementary material for: Mode of HIV acquisition among adolescents living with HIV in resource-limited settings: A data-driven approach from South Africa
Source: PLoS One. 2023 Feb 24;18(2):e0281298. doi: 10.1371/journal.pone.0281298 (PMC9955664; doi:10.1371/journal.pone.0281298)
Supplement: Attachment — Submitted filename: Review of MOHA in adolescents.docx [file pone.0281298.s001.docx]

**Mode of HIV acquisition among adolescents living with HIV in resource-limited settings: a data-driven approach from South Africa" Review**

**Is the rationale for the study clear and valid?**

Although the authors claim that there is a significant difference in the way that adolescents with HIV are treated dependent on whether their HIV was acquired from MTCT (vertical) or sexually acquired (horizontal), the case for this and the evidence was not compelling. It is not clear whether in resource-constrained environments of LMICs such as rural South Africa there can be different services. In the view of this reviewer for example adolescents with HIV need counselling around sexual and reproductive health and disclosure irrespective of how they acquired the HIV; adolescents need to be on ARTs for life and be virally suppressed irrespective of mode of acquisition.

So, the case for the paper is not clearly articulated.

**Statistics**

Although there are clear statistical numbers, viz Sensitivity, specificity and Area under the curve, it is not clear that any one age chosen between 10 and 15 will have much more practical difference on the identification of the mode of acquisition.

**Discussions**

The authors have not taken into account that MTCT of HIV is decreasing with the successful implementation of PMTCT programmes throughout SSA. As a result successive cohorts of adolescents will have less and less vertical transmission with sexual transmission being the main driver. The discussion also does not make clear case for how the mode of transmission affects clinical care in practice.

**Comprehension of language**

The scientific language is not always accurate. For example in background it states: “*The adolescent HIV incidence rate was 24% in 2018….”.*

Another example is the discussion is: “This also resulted in a delay in establishing the proper infrastructure to orchestrate such a feat”

Because this rationale is not made convincingly, the foundation for the paper is on shaky grounds.
