# Supplementary material for: Mode of HIV acquisition among adolescents living with HIV in resource-limited settings: A data-driven approach from South Africa
Source: PLoS One. 2023 Feb 24;18(2):e0281298. doi: 10.1371/journal.pone.0281298 (PMC9955664; doi:10.1371/journal.pone.0281298)
Supplement: Attachment — Submitted filename: PLOS ONE_Response to Reviewers.docx [file pone.0281298.s003.docx]

3 January 2023

To: The Editor, PLOS ONE

Responses to Editor comments

______________________________________________________________________________

*Note: We thank the editor for their comments and corrections on the manuscript. After careful revision, we have corrected the numbers for the cohort adolescents to reflect the correct sample size (n=1,107), and thus updated tables, text and figures throughout the manuscript where applicable. This can be seen in the track-changes version of the manuscript submitted.*

1. Background, first paragraph: sentence starting with ‘Further, facing the issues…’ appears to be incomplete.

*Thank you for this comment, we have altered the sentence structure to include ‘while simultaneously’ instead of ‘further’, to improve clarity.*

2. Background, second paragraph: provide reference for statement that the majority of current ALHIV is vertical transmission. Also, this seems to contradict the authors response to reviewers that in 2020 the numbers of vertically and sexually infected adolescents were about equal.

*This is a valid point, we have changed these sentences as indicated in point number 3.*

3. Background, second paragraph: reference to most infected children pass away within their first year is outdated. This was true of untreated children early on in the epidemic but is no longer true as many vertically infected children are now living to adolescence and adulthood with ART. Sentence needs to be revised.

*We have revised the sentence to read:*

*“Although the risk of early mortality is increased for vertically infected children who are not initiated on ARV, research has shown that approximately 30% of vertically infected adolescents remain asymptomatic for 10 to 16 years.”*

4. Background, last paragraph: add a period after refs [18, 19, 5].

*Thank you, a period has been added.*

5. Table 1: why does the title refer to N=1107 instead of N=1141?

*Thank you for this observation – the authors have addressed this inconsistency when updating the cohort sample size throughout the analysis.*

6. Table 1: mean/SD is calculated – confirm that data are normally distributed or change to median/IQR.

*The numbers have been changed to median and IQR for better accuracy.*

7. MOHA allocation and confirmation, first paragraph: first sentence refers to 69.6% categorized as vertically infected with age cutoff of 10. This doesn’t match Table 2 which has 69.9% for mothers/non-mothers combined.

*This error has been corrected in the revised sample size.*

8. MOHA allocation and confirmation, second paragraph: delete ‘the’ in first sentence (… we found that most adolescents were ….).

*Thank you for bringing this to our attention, the ‘the’ has been removed from the sentence.*

9. MOHA allocation and confirmation, third paragraph: first sentence refers to 90.7% of participants with sexually acquired HIV using age cutoff of 10 (original allocation). This doesn’t match Table 2 (88.8%).

*The paragraph data was correct, and the table has been updated to reflect the correct numbers.*

10. MOHA allocation and confirmation, third paragraph: 4.6% vertically infected vs. 4.7% in the Table 2.

*This was a rounding inconsistency. The table is correct, so the paragraph was edited to read ‘4.7%’.*

11. Table 3: add in N’s for sexually acquired vs. vertically acquired.

*N’s for totals have been added.*

12. MOHA allocation and confirmation, fourth paragraph: this paragraph refers to the adolescents, but I think should refer to the additional young mothers. Please revise as needed.

*Appreciation for picking up this unclarity. The text was edited here to clarify that this is referring to the additional young mothers, by replacing ‘adolescents’ with ‘young mothers’.*

13. Table 4: add in N’s for sexually acquired vs. vertically acquired.

*N’s for each branch have been added.*

14. Table 4: what does ‘Avg (SD) total agreement’ refer to?

*This refers to the average number of affirmative answers to each branch after filtering into the vertical or sexual tree. In other words, on average, how many of the logic tree questions did each participant answer ‘yes’ to. For clarity, the table heading has been changed to ‘Avg affirmative answers’.*

15. Determination of optimal cut-off age, first paragraph: delete duplicate ‘had’ in first sentence (… ART initiation at 10 years old had the highest sensitivity…).

*Thank you, the duplicate ‘had’ has been deleted.*

Responses to additional comments: 3 January 2023

1. Please state what role the funders took in the study. If the funders had no role, please state: "The funders had no role in study design, data collection and analysis, decision to publish, or preparation of the manuscript."

*The funders had no role in study design, data collection and analysis, decision to publish, or preparation of the manuscript.*

2. In the Methods section please revise the informed consent statement to reflect whether written/verbal informed consent was obtained from all participants for inclusion in the study.

*On Page 4 paragraph 2, the word “written” was added to clarify the type of informed consent obtained.*

*“Voluntary written informed consent was also obtained from these additional participants” was added to Page 5 paragraph 1 (for the additional young mothers).*
