# Supplementary material for: Mode of HIV acquisition among adolescents living with HIV in resource-limited settings: A data-driven approach from South Africa
Source: PLoS One. 2023 Feb 24;18(2):e0281298. doi: 10.1371/journal.pone.0281298 (PMC9955664; doi:10.1371/journal.pone.0281298)
Supplement: Attachment — Submitted filename: PONE-D-22-11073 Reviewer Comments 220523.docx [file pone.0281298.s002.docx]

**Manuscript Number PONE-D-22-11073:
"Mode of HIV acquisition among adolescents living with HIV in resource-limited settings: a data-driven approach from South Africa"**

**Reviewer Comments**

The article has both strengths and weaknesses which are summarised below:

Strengths

1. Understanding mode of HIV acquisition (MOHA) has relevance for planning and provision of HIV services, and the study based on a large cohort of adolescents provides information that is relevant from a population and epidemiological perspective.
2. The study is mostly methodologically sound (with some limitations as noted below).

Weaknesses

3. The article overstates the importance of mode of acquisition for individual patient care and support decisions. Such decisions should be based on individual patient need rather than on mode of acquisition. For example, whilst adolescents with vertically-acquired infection are at higher risk of experiencing cognitive delay and poor physical health, interventions aimed at mitigating the effects of these conditions, should be available to all adolescents, irrespective of MOHA.
4. The study’s aim and objective, findings and discussion/conclusions are not fully aligned, and the abstract and the article are likewise not fully congruent. For example, the article concludes with the statement that “incorporating MOHA in LMICs will also be necessary to reach the UNAIDS 90-90-90 goal as adolescent incidence rates are not decreasing as rapidly as other age groups and loss to follow-up remains high in these countries”. This is not supported by the content of the article, and a more modest conclusion based on the findings of this study would be more appropriate.
5. Some of the assumptions used in the study’s logic tree may not be justified, and may therefore limit its utility. In particular, it is problematic to assume that transmission is always sexually acquired, if the adolescent was sexually active before initiating ART. This should as a minimum be highlighted as a limitation.
6. Some of the statements about the health system do not make sense. For example, the statement that the study population included “adolescents who had ever initiated ART in a municipality in the Eastern Cape” is confusing, as there is no part of the Eastern Cape which is not in a municipality – or does it mean something else? It is not clear if the study includes only adolescents initiated in the public health sector, or whether (the presumably small number of) adolescents receiving care in the private sector are included.

Recommendation

Overall, I would recommend accepting the article providing that there is clearer alignment between and articulation of the aims, results, discussion and conclusion - both in the abstract and the text of the article. The article should focus on clearly reporting its finding related to methods for determining MOHA, and ensure that all conclusions are based on and fully justified by these findings.
